# Care of HIV Patients in Long-Term Care Facilities: A Growing Concern and a Call to Action

**DOI:** 10.26502/acmcr.96550727

**Published:** 2025-08-20

**Authors:** Minghsun Liu, William D. King, Derrick Butler, Mitchell H. Liu

**Affiliations:** 1 Assistant Professor, Prime West/Centinela Hospital Medical Center IM Program & California University of Science and Medicine, National Senior Medical Director, Woundtech, 11103 Venice Blvd., Los Angeles; 2 Associate Professor, Department of Internal Medicine, Charles R. Drew University of Medicine and Science; 3 Chief Medical Officer, T.H.E. Health and Wellness Centers; 4 Student, Brentwood School

**Keywords:** HIV, Long term care, aging and HIV, HIV in nursing homes, HIV stigma

## Abstract

As the population of people living with HIV (PWH) ages, they face increased risks of chronic diseases and may require care in nursing homes (NHs). This study identifies systemic barriers to optimal HIV care in NHs through three case examples, illustrating issues such as knowledge gaps in HIV management across the care spectrum, miscommunication during transition of care, and stigma. Proposed solutions include targeted education, improved drug interaction software, and enhanced protocols for HIV care in NHs. Addressing these gaps is crucial for improving outcomes for this aging and vulnerable population.

## Introduction

Persons living with HIV (PWH) are living longer. HIV prevalence data from 2021 shows 63.3% of PWH are over 45 years-old (and 40.6% are aged 55+) (1). The projection is that by 2030, over 70% of PLWH will be older than 50 years (2). As PWH ages, they face onset of inflammation and the increased risk of chronic diseases such as cardiovascular disease, diabetes, renal disease and musculoskeletal afflictions. These conditions could result in the need for temporary or permanent care in nursing homes (NH). HIV has become more manageable with single tablet regimens and long acting injectables. However, NH teams may lack the specialized training to address HIV care, leading to care gaps for PWH at NH settings. Literature suggests most NH do not provide HIV/AIDS specialty care (3) and challenges in care range from lower quality of care (4); to worse outcomes for PWH in NH compared to those not living with HIV (5). We have identified three cases of PWH hospitalized from NH to highlight some of the care gaps for PWH in NH settings. Our goal is not to critique the qualification of NH teams or the qualities of NH facilities but to highlight the need for systemic changes in education and institutional protocols around HIV care in NHs.

### Case 1:

A 63-year-old male was readmitted to a NH after recurrent hospitalizations. The patient’s medication reconciliation was flagged by the NH pharmacy for duplicate therapy, noting that “Prezista (darunavir) and ritonavir have the same mechanism of action (protease inhibitor).” Investigation showed that darunavir and ritonavir were the only antiretrovirals on the medication reconciliation. Prior to the last hospitalization, his regimen was listed as lamivudine (3TC), darunavir/ritonavir (DRV/r). Records from three years prior showed he was on abacavir (ABC), 3TC, DRV/r. His last CD4 count was 577 cells/μL and 13% with an undetectable viral load. The patient was restarted on ABC/3TC/DRV/r with follow-up labs and appointment orders. The lead pharmacist later clarified that the alert should not have been sent to providers, but did not specify which drug reference system generated the alert.

### Case 2:

A 57-year-old male was admitted to a NH after a severe motor vehicle accident with multiple fractures and injuries leading to impaired memory. The NH staff was aware he had been followed by a local clinic but was not aware the clinic serves PWH. It was not until three months later when an alert NH staff member noticed the clinic name and realized the patient had been living with HIV. The patient was restarted on his antiretrovirals after additional records were obtained. His CD4 count was 461 cells/μL and 29% with a viral load of 148,339 copies when he resumed his antiretroviral therapy (ART).

### Case 3:

A 54-year-old female living with HIV was admitted from a NH with fever and pneumonia. Her medication reconciliation showed she was only on 3TC and lopinavir/ritonavir (LPV/r). She had been seen in the same hospital three times over the course of four years where her ART was always documented as only 3TC and LPV/r. The very first note from four years ago mentioned the patient’s HIV was under control with a CD4 count over 500 cells/μL and an undetectable viral load. By the most recent admission, her CD4 count was 89 cells/μL and 26%, with a viral load of 756 copies. She was started on trimethoprim/sulfamethoxazole (TMP/SMX) prophylaxis, and viral load and genotype testing were ordered. She was started on tenofovir disoproxil fumarate/emtricitabine/dolutegravir (TDF/FTC/DTG) via G-tube at the time of discharge with follow-up scheduled.

Barriers and gaps that we have identified based on the presented cases are:

- Due to the high cost of current co-formulated HIV medications, many inpatient pharmacies, particularly community-based ones, are unable to provide the combination pills. Instead, they are provided as individual components. This introduces complexity (multiple medications being reconciled into one single medication or vice versa during admissions/discharges) and confusion (due to lack of provider knowledge about appropriate combinations and/or substitutions of HIV medications).
- In a NH setting, PWH, even without regular visits for HIV care, might continue to receive antiretroviral regimens, including inadequate ones, often under the false impression that “something is better than nothing.” It is insufficient for NH teams to recognize the importance of PWH taking HIV medications; it is also critical for providers to recognize inappropriate/inadequate ART regimens for escalation and further review.
- Drug-checking software used by the general public as well as pharmacies needs to be validated with regard to the appropriate classification of ritonavir (and cobicistat). We surveyed five online drug-checking websites (four available to general consumers and one healthcare provider-oriented/restricted). None of them marked ritonavir or cobicistat as duplicate therapy with any of the protease inhibitors (PIs) we assessed. However, the results from consumer-oriented websites are not uniformly satisfactory. One website consistently flagged multiple ritonavir+PI or cobicistat+PI combinations as “Serious - Use Alternative.” A second site also gives inconsistent warnings depending on the order of the two drugs. Most sites flag many of the boosted-PI combinations as “SERIOUS/MAJOR.” The website restricted to healthcare providers consistently has more accurate and nuanced discussions about the combinations, referencing the use of ritonavir and cobicistat specifically as boosting agents in available combinations (6, 7, 8, Additional Material).
- Finally, we do not consider this a true care gap but more of a reflection of the historical stigma associated with HIV infection. Many well-established HIV clinics either use euphemisms or do not mention HIV directly in their names. This does pose additional challenges during patient intakes.

These barriers can potentially undo years of excellent work by the medical community in reversing the devastating impact of HIV infections across the U.S. Possible approaches to address barriers identified above include:

- Targeted education during training (medical, pharmacy, nurse practitioner, and physician assistant schools; emergency, family, and internal medicine, as well as pharmacy residencies) plus continuing education about infections that require cocktail therapies (e.g., HIV, active tuberculosis) and basic awareness about what constitutes appropriate regimens for these infections.
- Enhancements to drug interaction-checking software to better recognize combination therapies. Disclaimers or prompts should be added to ensure that providers verify the completeness of prescribed regimens.
- Implement pharmacy protocols to escalate medications that are often part of combination therapies for infections mentioned above to ensure appropriate, complete regimens are being dispensed for the correct diagnoses (PrEP vs. HIV infection, or latent TB vs. active TB).
- Mandate HIV screening tests or verification of HIV status with the local department of health as a part of NH intake processes. The mandate would be in line with current recommendations on HIV screening from the CDC. (9).

PWH living in NH settings can be particularly vulnerable to receiving suboptimal HIV care. The cases presented highlight the need for systemic changes, including improved education, enhanced drug interaction software, and better protocols for the care of PWH in NH settings. Addressing these gaps is essential to improving outcomes for this aging and vulnerable population.

## Supplementary Material

Evaluation of Online Drug Interaction Checkers

Additional Material:

“Evaluation of Online Drug Interaction Checkers” Excel Sheet. Accessed February 4th, 2025.

## References

[1] HIV Prevalence (nd) AIDSVu.

[2] Ford CL, Wallace SP, Newman PA, Belief in AIDS-Related Conspiracy Theories and Mistrust in the Government: Relationship with HIV Testing Among At-Risk Older Adults. The Gerontologist 57 (2017): S48–S58. 10.1093/geront/gns192 PMC3826163 23362210

[3] Greene M & Justice AC. Understanding the Relationship between Nursing Home Experience with HIV and Patient Outcomes. Open Forum Infectious Diseases 5 (2018): S284.

[4] Bozzette SA, Berry SH, Duan N, The care of HIV-infected adults in rural areas of the United States. Journal of acquired immune deficiency syndromes and human retrovirology: official publication of the International Retrovirology Association 20 (1999): 193–198.

[5] Greene M, & Justice AC. Quality of nursing homes that serve patients with human immunodeficiency virus. SAGE Open Medicine 5 (2017): 205031211772635.

[6] Protease inhibitors: A healthcare professional guide to HIV drugs (2022).

[7] Everything you need to know about protease inhibitors (2018).

[8] Protease Inhibitors for HIV Infection. (n.d.). WebMD.

[9] Branson BM, Handsfield HH, Lampe MA, Janssen RS, Centers for Disease Control and Prevention (CDC). Revised recommendations for HIV testing of adults, adolescents, and pregnant women in health-care settings. MMWR Recomm Rep 55 (2006): 1–17. 16988643

