# Supplementary material for: Care of HIV Patients in Long-Term Care Facilities: A Growing Concern and a Call to Action
Source: Arch Clin Med Case Rep. Author manuscript; Available in PMC 2025 Sep 5. (PMC12410836; doi:10.26502/acmcr.96550727)
Supplement: Evaluation of Online Drug Interaction Checkers [file NIHMS2106823-supplement-Evaluation_of_Online_Drug_Interaction_Checkers.pdf]

Drug Interaction Checkers	Protease Inhibitor	Drugs Interactions (with ritonavir)	Drugs Interactions (with cobcistat)
https://reference.medscape.com/drug-interactionchecker	Amprenavir.	N/A	N/A
	Atazanavir.	Monitor Closely<br>atazanavir + ritonavir<br>atazanavir will increase the level or effect of ritonavir by affecting hepatic/intestinal enzyme CYP3A4 metabolism. Use Caution/Monitor.<br><br>atazanavir + ritonavir<br>atazanavir and ritonavir both increase risk of immune reconstitution syndrome. Use Caution/Monitor.<br><br>Minor<br>ritonavir + atazanavir<br>ritonavir will increase the level or effect of atazanavir by affecting hepatic/intestinal enzyme CYP3A4 metabolism. Minor/Significance Unknown.	Serious - Use Alternative<br>atazanavir + cobicistat<br>atazanavir will increase the level or effect of cobicistat by affecting hepatic/intestinal enzyme CYP3A4 metabolism. Avoid or Use Alternate Drug.<br><br>cobicistat + atazanavir<br>cobicistat will increase the level or effect of atazanavir by affecting hepatic/intestinal enzyme CYP3A4 metabolism. Avoid or Use Alternate Drug.
	Darunavir.	Serious - Use Alternative<br>ritonavir + darunavir<br>ritonavir will increase the level or effect of darunavir by affecting hepatic/intestinal enzyme CYP3A4 metabolism. Avoid or Use Alternate Drug.<br><br>Monitor Closely<br>darunavir + ritonavir<br>darunavir will increase the level or effect of ritonavir by affecting hepatic/intestinal enzyme CYP3A4 metabolism. Use Caution/Monitor.<br><br>darunavir + ritonavir<br>darunavir and ritonavir both increase risk of immune reconstitution syndrome. Use Caution/Monitor.	Serious - Use Alternative<br>cobicistat + darunavir<br>cobicistat will increase the level or effect of darunavir by affecting hepatic/intestinal enzyme CYP3A4 metabolism. Avoid or Use Alternate Drug.<br><br>darunavir + cobicistat<br>darunavir will increase the level or effect of cobicistat by affecting hepatic/intestinal enzyme CYP3A4 metabolism. Avoid or Use Alternate Drug.
	Indinavir.	Monitor Closely<br>indinavir + ritonavir<br>indinavir will increase the level or effect of ritonavir by affecting hepatic/intestinal enzyme CYP3A4 metabolism. Use Caution/Monitor.<br><br>ritonavir + indinavir<br>ritonavir will increase the level or effect of indinavir by affecting hepatic/intestinal enzyme CYP3A4 metabolism. Use Caution/Monitor.<br><br>indinavir + ritonavir<br>indinavir will increase the level or effect of ritonavir by P-glycoprotein (MDR1) efflux transporter. Use Caution/Monitor.<br><br>indinavir + ritonavir<br>indinavir and ritonavir both increase risk of immune reconstitution syndrome. Use Caution/Monitor.	Contraindicated<br>cobicistat + indinavir<br>cobicistat will increase the level or effect of indinavir by affecting hepatic/intestinal enzyme CYP3A4 metabolism. Contraindicated. Contraindicated with cobicistat coadministered with atazanavir only; both atazanavir and indinavir are associated with indirect (unconjugated) hyperbilirubinemia<br><br>Serious - Use Alternative<br>indinavir + cobicistat<br>indinavir will increase the level or effect of cobicistat by affecting hepatic/intestinal enzyme CYP3A4 metabolism. Contraindicated.
	Fosamprenavir.	Serious - Use Alternative<br>ritonavir + fosamprenavir<br>ritonavir will increase the level or effect of fosamprenavir by affecting hepatic/intestinal enzyme CYP3A4 metabolism. Avoid or Use Alternate Drug.<br><br>Monitor Closely<br>fosamprenavir + ritonavir<br>fosamprenavir will increase the level or effect of ritonavir by affecting hepatic/intestinal enzyme CYP3A4 metabolism. Use Caution/Monitor.<br><br>fosamprenavir + ritonavir<br>fosamprenavir and ritonavir both increase risk of immune reconstitution syndrome. Use Caution/Monitor.	No Interactions Found
	Lopinavir.	Monitor Closely<br>ritonavir + lopinavir<br>ritonavir will increase the level or effect of lopinavir by affecting hepatic/intestinal enzyme CYP3A4 metabolism. Use Caution/Monitor.	No interactions Found
	Nelfinavir.	Monitor Closely<br>nelfinavir + ritonavir<br>nelfinavir will increase the level or effect of ritonavir by affecting hepatic/intestinal enzyme CYP3A4 metabolism. Use Caution/Monitor.<br><br>ritonavir + nelfinavir<br>ritonavir will increase the level or effect of nelfinavir by affecting hepatic/intestinal enzyme CYP3A4 metabolism. Use Caution/Monitor.<br><br>ritonavir + nelfinavir<br>ritonavir will increase the level or effect of nelfinavir by P-glycoprotein (MDR1) efflux transporter. Use Caution/Monitor.<br><br>nelfinavir + ritonavir<br>nelfinavir and ritonavir both increase risk of immune reconstitution syndrome. Use Caution/Monitor.	No interactions Found
	Saquinavir.	Serious - Use Alternative<br>saquinavir + ritonavir<br>saquinavir will increase the level or effect of ritonavir by affecting hepatic/intestinal enzyme CYP3A4 metabolism. Avoid or Use Alternate Drug.<br><br>Monitor Closely<br>ritonavir + saquinavir<br>ritonavir will increase the level or effect of saquinavir by affecting hepatic/intestinal enzyme CYP3A4 metabolism. Use Caution/Monitor.<br><br>ritonavir + saquinavir<br>ritonavir will increase the level or effect of saquinavir by P-glycoprotein (MDR1) efflux transporter. Use Caution/Monitor.<br><br>ritonavir + saquinavir<br>ritonavir and saquinavir both increase risk of immune reconstitution syndrome. Use Caution/Monitor.	Serious - Use Alternative<br>cobicistat + saquinavir<br>cobicistat will increase the level or effect of saquinavir by affecting hepatic/intestinal enzyme CYP3A4 metabolism. Avoid or Use Alternate Drug.<br><br>cobicistat + saquinavir<br>cobicistat will increase the level or effect of saquinavir by P-glycoprotein (MDR1) efflux transporter. Avoid or Use Alternate Drug.<br><br>saquinavir + cobicistat<br>saquinavir will increase the level or effect of cobicistat by affecting hepatic/intestinal enzyme CYP3A4 metabolism. Contraindicated.
	Tipranavir.	Monitor Closely<br>ritonavir + tipranavir<br>ritonavir will increase the level or effect of tipranavir by affecting hepatic/intestinal enzyme CYP3A4 metabolism.<br><br>ritonavir + tipranavir<br>ritonavir and tipranavir both increase risk of immune reconstitution syndrome. Use Caution/Monitor.	Contraindicated<br>cobicistat + tipranavir<br>cobicistat will increase the level or effect of tipranavir by affecting hepatic/intestinal enzyme CYP3A4 metabolism. Contraindicated.<br><br>Serious - Use Alternative<br>tipranavir + cobicistat<br>tipranavir will increase the level or effect of cobicistat by affecting hepatic/intestinal enzyme CYP3A4 metabolism. Contraindicated.
https://www.webmd.com/interaction-checker/default.htm	Amprenavir.	N/A	N/A
	Atazanavir.	MONITOR CLOSELY<br>Significant interaction possible (monitoring by your doctor required).<br><br>Atazanavir + Ritonavir<br>Atazanavir will increase the level or effect of Ritonavir by altering drug metabolism.<br><br>Atazanavir and Ritonavir both increase risk of immune reconstitution syndrome<br>MINOR<br>Interaction is unlikely, minor, or nonsignificant.<br><br>Ritonavir + Atazanavir<br>Ritonavir will increase the level or effect of Atazanavir by altering drug metabolism.	SERIOUS<br>Potential for serious interaction; regular monitoring by your doctor required or alternate medication may be needed.<br><br>Atazanavir + Cobicistat<br>Atazanavir will increase the level or effect of Cobicistat by altering drug metabolism.Atazanavir will increase the level or effect of Cobicistat by altering drug metabolism.<br>Atazanavir will increase the level or effect of Cobicistat by altering drug metabolism.Atazanavir will increase the level or effect of Cobicistat by altering drug metabolism.
	Darunavir.	SERIOUS<br>Potential for serious interaction; regular monitoring by your doctor required or alternate medication may be needed.<br><br>Ritonavir + Darunavir<br>Ritonavir will increase the level or effect of Darunavir by altering drug metabolism.	SERIOUS<br>Potential for serious interaction; regular monitoring by your doctor required or alternate medication may be needed.<br><br>Darunavir + Cobicistat<br>Darunavir will increase the level or effect of Cobicistat by altering drug metabolism.Darunavir will increase the level or effect of Cobicistat by altering drug metabolism.<br>Darunavir will increase the level or effect of Cobicistat by altering drug metabolism.Darunavir will increase the level or effect of Cobicistat by altering drug metabolism.
	Indinavir.	MONITOR CLOSELY<br>Significant interaction possible (monitoring by your doctor required).<br><br>Indinavir + Ritonavir<br>Indinavir will increase the level or effect of Ritonavir by altering drug metabolism.<br><br>Indinavir will increase the level or effect of Ritonavir by affects how the drug is eliminated from the body<br><br>Indinavir and Ritonavir both increase risk of immune reconstitution syndrome<br>Indinavir will increase the level or effect of Ritonavir by altering drug metabolism.	DON'T USE TOGETHER<br>Never use this combination of drugs because of high risk for dangerous interaction.<br><br>Cobicistat + Indinavir<br>Cobicistat will increase the level or effect of Indinavir by altering drug metabolism.<br><br>Additional Information: Cobicistat increases levels or effect of indinavir. This combination should be avoided.<br>SERIOUS<br>Potential for serious interaction; regular monitoring by your doctor required or alternate medication may be needed.<br><br>Indinavir + Cobicistat<br>Indinavir will increase the level or effect of Cobicistat by altering drug metabolism.

	Fosamprenavir.	SERIOUS<br>Potential for serious interaction; regular monitoring by your doctor required or alternate medication may be needed.<br><br>Ritonavir + Fosamprenavir<br>Ritonavir will increase the level or effect of Fosamprenavir by altering drug metabolism.<br><br>MONITOR CLOSELY<br>Significant interaction possible (monitoring by your doctor required).<br><br>Fosamprenavir + Ritonavir<br>Fosamprenavir will increase the level or effect of Ritonavir by altering drug metabolism.<br><br>Fosamprenavir and Ritonavir both increase risk of immune reconstitution syndrome	No Interactions Found
	Lopinavir.	MONITOR CLOSELY<br>Significant interaction possible (monitoring by your doctor required).<br><br>Ritonavir + Lopinavir<br>Ritonavir will increase the level or effect of Lopinavir by altering drug metabolism.	No Interactions Found
	Nelfinavir.	MONITOR CLOSELY<br>Significant interaction possible (monitoring by your doctor required).<br><br>Nelfinavir + Ritonavir<br>Nelfinavir will increase the level or effect of Ritonavir by altering drug metabolism.<br><br>Nelfinavir and Ritonavir both increase risk of immune reconstitution syndrome<br>Nelfinavir will increase the level or effect of Ritonavir by altering drug metabolism.<br><br>Nelfinavir will increase the level or effect of Ritonavir by affects how the drug is eliminated from the body	No Interactions Found
	Saquinavir.	SERIOUS<br>Potential for serious interaction; regular monitoring by your doctor required or alternate medication may be needed.<br><br>Saquinavir + Ritonavir<br>Saquinavir will increase the level or effect of Ritonavir by altering drug metabolism.<br><br>MONITOR CLOSELY<br>Significant interaction possible (monitoring by your doctor required).<br><br>Ritonavir + Saquinavir<br>Ritonavir will increase the level or effect of Saquinavir by altering drug metabolism.<br><br>Additional Information: Saquinavir is usually combined with ritonavir. The combination may increase side effects of saquinavir.<br><br>Ritonavir will increase the level or effect of Saquinavir by affects how the drug is eliminated from the body<br><br>Ritonavir and Saquinavir both increase risk of immune reconstitution syndrome	SERIOUS<br>Potential for serious interaction; regular monitoring by your doctor required or alternate medication may be needed.<br><br>Saquinavir + Cobicistat<br>Saquinavir will increase the level or effect of Cobicistat by altering drug metabolism.<br><br>Saquinavir will increase the level or effect of Cobicistat by altering drug metabolism.<br><br>Saquinavir will increase the level or effect of Cobicistat by affects how the drug is eliminated from the body<br>Saquinavir will increase the level or effect of Cobicistat by altering drug metabolism.<br><br>Saquinavir will increase the level or effect of Cobicistat by altering drug metabolism.<br><br>Saquinavir will increase the level or effect of Cobicistat by affects how the drug is eliminated from the body (via what is known as the P-glycoprotein [MDR1] transporter).<br>Saquinavir will increase the level or effect of Cobicistat by altering drug metabolism.<br><br>Saquinavir will increase the level or effect of Cobicistat by altering drug metabolism.<br><br>Saquinavir will increase the level or effect of Cobicistat by affects how the drug is eliminated from the body (via what is known as the P-glycoprotein [MDR1] transporter).
	Tipranavir.	MONITOR CLOSELY<br>Significant interaction possible (monitoring by your doctor required).<br><br>Ritonavir + Tipranavir<br>Ritonavir will increase the level or effect of Tipranavir by altering drug metabolism.<br><br>Additional Information: Taking tipranavir with ritonavir combined is essential for therapy.<br><br>Ritonavir and Tipranavir both increase risk of immune reconstitution syndrome	DON'T USE TOGETHER<br>Never use this combination of drugs because of high risk for dangerous interaction.<br><br>Cobicistat + Tipranavir<br>Cobicistat will increase the level or effect of Tipranavir by altering drug metabolism.<br><br>SERIOUS<br>Potential for serious interaction; regular monitoring by your doctor required or alternate medication may be needed.<br><br>Tipranavir + Cobicistat<br>Tipranavir will increase the level or effect of Cobicistat by altering drug metabolism.
https://www.drugs.com/interaction/list/?drug_list=2021-0	Amprenavir.	N/A	N/A
	Atazanavir.	Moderate<br>ritonavir atazanavir<br>Applies to: ritonavir, atazanavir<br><br>Using atazanavir together with ritonavir may increase the effects of atazanavir.	No interactions Found
	Darunavir.	No interactions Found	No interactions Found
	Indinavir.	Moderate<br>ritonavir indinavir<br>Applies to: ritonavir, indinavir<br><br>Using indinavir together with ritonavir may increase the effects of indinavir.<br><br>Switch to professional interaction data	Moderate<br>indinavir cobicistat<br>Applies to: indinavir, cobicistat<br><br>Cobicistat is a medication used to boost the effects of some medications that treat HIV infection. However, using cobicistat together with other HIV medications<br>Drug and food interactions
	Fosamprenavir.	No interactions Found	Moderate<br>fosamprenavir cobicistat<br>Applies to: fosamprenavir, cobicistat<br><br>Cobicistat is a medication used to boost the effects of some medications that treat HIV infection. However, using cobicistat together with other HIV medications
	Lopinavir.	No interactions Found	
	Nelfinavir.	Moderate<br>ritonavir nelfinavir<br>Applies to: ritonavir, nelfinavir<br><br>Using nelfinavir together with ritonavir may increase the effects of nelfinavir. Drug and food interactions	Moderate<br>nelfinavir cobicistat<br>Applies to: nelfinavir, cobicistat<br><br>Cobicistat is a medication used to boost the effects of some medications that treat HIV infection. However, using cobicistat together with other HIV medications
	Saquinavir.	Moderate<br>saquinavir ritonavir<br>Applies to: saquinavir, ritonavir<br><br>Using saquinavir together with ritonavir may increase the effects of saquinavir. Switch to professional interaction data	Moderate<br>saquinavir cobicistat<br>Applies to: saquinavir, cobicistat<br><br>Cobicistat is a medication used to boost the effects of some medications that treat HIV infection. However, using cobicistat together with other HIV medications
	Tipranavir.	Major<br>ritonavir tipranavir<br>Applies to: ritonavir, tipranavir<br><br>Using ritonavir and tipranavir can cause serious side effects that may affect your liver.<br>Drug and food interactions	Moderate<br>tipranavir cobicistat<br>Applies to: tipranavir, cobicistat<br><br>Cobicistat is a medication used to boost the effects of some medications that treat HIV infection. However, using cobicistat together with other HIV medications
https://go.drugbank.com/drug-interaction-checker#results	Amprenavir.	N/A	N/A
	Atazanavir.	No interactions Found	No interactions Found
	Darunavir.	No interactions Found	No interactions Found
	Indinavir.	Ritonavir<br>Indinavir<br>SEVERITY<br>MAJOR<br>DESCRIPTION<br>The serum concentration of Indinavir can be increased when it is combined with Ritonavir.<br><br>EXTENDED DESCRIPTION<br>Prescribing information for both ritonavir and indinavir states that their concurrent use should be avoided	Indinavir<br>Cobicistat<br>SEVERITY<br>MAJOR<br>DESCRIPTION<br>The metabolism of Cobicistat can be decreased when combined with Indinavir.<br><br>EXTENDED DESCRIPTION<br>The subject drug is a strong CYP3A4 inhibitor, and the affected drug is metabolized by CYP3A4. Concomitant administration will decrease the metabolism of the affected drug, increasing serum concentrations

	Fosamprenavir.	Ritonavir<br>Fosamprenavir<br>SEVERITY<br>MINOR<br>DESCRIPTION<br>The serum concentration of amprenavir, an active metabolite of Fosamprenavir, can be increased when used in combination with Ritonavir.<br><br>EXTENDED DESCRIPTION<br>The co-administration of fosamprenavir with ritonavir can lead to increased serum concentrations of fosamprenavir's active metabolite, amprenavir.	Cobicistat<br>Fosamprenavir<br>SEVERITY<br>MAJOR<br>DESCRIPTION<br>The metabolism of Fosamprenavir can be decreased when combined with Cobicistat.<br><br>EXTENDED DESCRIPTION<br>The subject drug is a strong CYP3A4 inhibitor, and the affected drug is metabolized by CYP3A4. Concomitant administration will decrease the metabolism of the affected drug, increasing serum concentrations
	Lopinavir.	No interactions Found	Lopinavir / Ritonavir<br>Cobicistat<br>SEVERITY<br>MAJOR<br>DESCRIPTION<br>The metabolism of Cobicistat can be decreased when combined with Lopinavir.<br><br>EXTENDED DESCRIPTION<br>The subject drug is a strong CYP3A4 inhibitor, and the affected drug is metabolized by CYP3A4. Concomitant administration will decrease the metabolism of the affected drug, increasing serum concentrations, as well as the risk and severity of adverse effects.1,2,3<br><br>REFERENCES<br>Zhou SF: Drugs behave as substrates, inhibitors and inducers of human cytochrome P450 3A4. Curr Drug Metab. 2008 May;9(4):310-22. [Article]<br>Lynch T, Price A: The effect of cytochrome P450 metabolism on drug response, interactions, and adverse effects. Am Fam Physician. 2007 Aug 1;76(3):391-6. [Article]<br>Klein K, Zanger UM: Pharmacogenomics of Cytochrome P450 3A4: Recent Progress Toward the "Missing Heritability" Problem. Front Genet. 2013 Feb 25;4:12. doi: 10.3389/fgene.2013.00012. eCollection 2013. [Article]<br>Lopinavir / Ritonavir<br>Cobicistat<br>SEVERITY<br>MODERATE<br>DESCRIPTION<br>The serum concentration of Cobicistat can be increased when it is combined with Ritonavir.<br><br>EXTENDED DESCRIPTION<br>Ritonavir is a strong inhibitor of CYP3A4.1 Its co-administration with substrates of CYP3A4, such as the affected drug, can lead to elevated serum concentrations of the 3A4 substrate. As ritonavir is a strong inhibitor, elevations in serum concentration may be significant and may increase the risk of serious adverse effects and toxicities.<br><br>REFERENCES<br>FDA Approved Drug Products: Norvir (ritonavir) for oral use [Link]
	Nelfinavir.	Ritonavir<br>Nelfinavir<br>SEVERITY<br>MODERATE<br>DESCRIPTION<br>The serum concentration of Nelfinavir can be increased when it is combined with Ritonavir.<br><br>EXTENDED DESCRIPTION<br>Ritonavir is a strong inhibitor of CYP3A4.1 Its co-administration with substrates of CYP3A4, such as the affected drug, can lead to elevated serum	Nelfinavir<br>Cobicistat<br>SEVERITY<br>MAJOR<br>DESCRIPTION<br>The metabolism of Cobicistat can be decreased when combined with Nelfinavir.<br><br>EXTENDED DESCRIPTION<br>The subject drug is a strong CYP3A4 inhibitor, and the affected drug is metabolized by CYP3A4. Concomitant administration will decrease the metabolism of the affected drug, increasing serum concentrations
	Saquinavir.	Ritonavir<br>Saquinavir<br>SEVERITY<br>MINOR<br>DESCRIPTION<br>The serum concentration of Saquinavir can be increased when it is combined with Ritonavir.<br><br>EXTENDED DESCRIPTION<br>Saquinavir must be administered in combination with ritonavir2 - as a potent inhibitor of multiple CYP enzymes,1 ritonavir helps to "boost" serum co	Saquinavir<br>Cobicistat<br>SEVERITY<br>MAJOR<br>DESCRIPTION<br>The metabolism of Cobicistat can be decreased when combined with Saquinavir.<br><br>EXTENDED DESCRIPTION<br>The subject drug is a strong CYP3A4 inhibitor, and the affected drug is metabolized by CYP3A4. Concomitant administration will decrease the metabolism of the affected drug, increasing serum concentrations
	Tipranavir.	Ritonavir<br>Tipranavir<br>SEVERITY<br>MINOR<br>DESCRIPTION<br>The serum concentration of Tipranavir can be increased when it is combined with Ritonavir.<br><br>EXTENDED DESCRIPTION<br>Tipranavir is a protease inhibitor which is always given in combination with ritonavir2 - the CYP enzyme inhibitory activity of ritonavir helps to "boost"	Tipranavir<br>Cobicistat<br>SEVERITY<br>MAJOR<br>DESCRIPTION<br>The metabolism of Cobicistat can be decreased when combined with Tipranavir.<br><br>EXTENDED DESCRIPTION<br>The subject drug is a strong CYP3A4 inhibitor, and the affected drug is metabolized by CYP3A4. Concomitant administration will decrease the metabolism of the affected drug, increasing serum concentrations
epocrates Web MultiCheck	Amprenavir.	N/A	N/A
	Atazanavir.	Monitor/Modify Tx<br>atazanavir + ritonavir<br><br>see atazanavir Adult/Peds Dosing for specific combo doses used for therapeutic advantage; monitor HR, ECG: ritonavir boosts atazanavir levels, e	Avoid/Use Alternative<br>atazanavir + cobicistat<br><br>avoid combo when cobicistat part of quadruple tx that is coformulated as complete regimen, otherwise use for therapeutic advantage: combo may incr. levels of both drugs, risk of adverse effects (hepatic metab. inhibited, duplicate tx)
	Darunavir.	Monitor/Modify Tx<br>darunavir + ritonavir<br><br>see darunavir Adult/Peds Dosing for specific combo doses used for therapeutic advantage; avoid combo when ritonavir is given as part of fixed-dos	No interactions Found
	Indinavir.	N/A	N/A
	Fosamprenavir.	Monitor/Modify Tx<br>fosamprenavir + ritonavir<br><br>see fosamprenavir Adult/Peds Dosing for specific combo doses used for therapeutic advantage: ritonavir boosts fosamprenavir levels, efficacy (hep	Avoid/Use Alternative<br>cobicistat + fosamprenavir<br><br>avoid combo: combo may not achieve adequate fosamprenavir levels, decr. efficacy; may decr. cobicistat levels, efficacy (hepatic metab. inhibited, cobicistat boosting may not be sufficient; hepatic metab. induced)
	Lopinavir.	Not applicable (already in a combo)	Avoid/Use Alternative<br>cobicistat + lopinavir/ ritonavir<br><br>avoid combo: combo may incr. cobicistat levels, risk of adverse effects (hepatic metab. inhibited; similar effects on CYP3A4)
	Nelfinavir.	Caution Advised<br>nelfinavir + ritonavir<br><br>caution advised, doses not established for combo use: combo may incr. nelfinavir levels, risk of adverse effects (hepatic metab. inhibited)	Avoid/Use Alternative<br>cobicistat + nelfinavir<br><br>avoid combo: combo may incr. cobicistat levels, risk of adverse effects (hepatic metab. inhibited)
	Saquinavir.	Monitor/Modify Tx<br>ritonavir + saquinavir<br><br>see saquinavir Adult/Peds Dosing for specific combo doses used for therapeutic advantage; monitor HR, consider monitoring ECG:	Avoid/Use Alternative<br>cobicistat + saquinavir<br><br>avoid combo: combo may not achieve adequate saquinavir levels, decr. efficacy; may incr. cobicistat levels, risk of adverse effects (hepatic metab. inhibited, cobicistat boosting may not be sufficient)
	Tipranavir.	Monitor/Modify Tx<br>ritonavir + tipranavir<br><br>see tipranavir Adult/Peds Dosing for specific combo doses used for therapeutic advantage: ritonavir boosts tipranavir levels, efficacy (hepatic metal	Avoid/Use Alternative<br>cobicistat + tipranavir<br><br>avoid combo: combo may not achieve adequate tipranavir levels, decr. efficacy; may incr. cobicistat levels, risk of adverse effects (hepatic metab. inhibited, cobicistat boosting may not be sufficient)
